# Antibacterial, antioxidant, and scolicidal activity investigation of biosynthesized ZnO NPs using *Zhumeria majdae* essential oil and hydroalcoholic extract

**DOI:** 10.1039/d5ra01192b

**Published:** 2025-07-08

**Authors:** Rana Kiani, Zahra Rafiee, Damoun Razmjoue, Ahmad Oryan, Mehrorang Ghaedi, Hassan Abidi

**Affiliations:** a Department of Chemistry, Yasouj University Yasouj 75918-74831 Islamic Republic of Iran z.rafiee@yu.ac.ir zahrarafiee2004@yahoo.com +98-741-222-3048 +98-741-222-3048; b Medicinal Plants Research Center, Yasuj University of Medical Sciences Yasuj Iran d.razmjoue@gmail.com; c Department of Pathology, School of Veterinary Medicine, Shiraz University Shiraz Iran; d Cellular and Molecular Research Center, Yasuj University of Medical Sciences Yasuj Iran

## Abstract

The biosynthesis of zinc oxide nanoparticles (ZnO NPs) from the *Zhumeria majdae* plant is reported in this study. Additionally, the essential oil and hydroalcoholic extract of this plant were manufactured and their antibacterial, antioxidant, and scolicidal activities were investigated. The essential oil was analyzed using GC/MS spectroscopy. The UV-vis spectrum of ZnO NPs exhibited an absorption maximum at 368 nm. The XRD and EDS confirmed the hexagonal structure of ZnO NPs and the presence of zinc. FE-SEM indicated well-defined cubic NPs. The FT-IR spectrum of ZnO NPs revealed the presence of functional groups, including flavonoids and polyphenolic biomolecules. The antibacterial activity of biosynthesized ZnO NPs, essential oil, and hydroalcoholic extract against *Staphylococcus aureus* and *Pseudomonas aeruginosa* was evaluated. Additionally, the scolicidal activity of various concentrations of these treatment regimens was investigated on protoscoleces of hydatid cysts at different time intervals. Furthermore, the antioxidant activity of these products was assessed using DPPH and FRAP tests. The antimicrobial effects of ZnO NPs, hydroalcoholic extract, and essential oil on *Staphylococcus aureus* and *Pseudomonas aeruginosa* were confirmed. The treatments demonstrated significant effects on protoscolexes of hydatid cysts at various time intervals, and their lethal effects were validated. From the findings of this study, it is believed that the essential oil of the *Zhumeria majdae* plant is more effective on some species of bacteria and also has significant scolicidal properties.

## Abstract 2

ZnO NPs was synthesized by green synthesis method, using *Zhumeria majdae* plant and it was characterized. The essential oil and hydroalcoholic extract were made and their antibacterial, antioxidant, and scolicidal activities were investigated.

## Graphical abstract

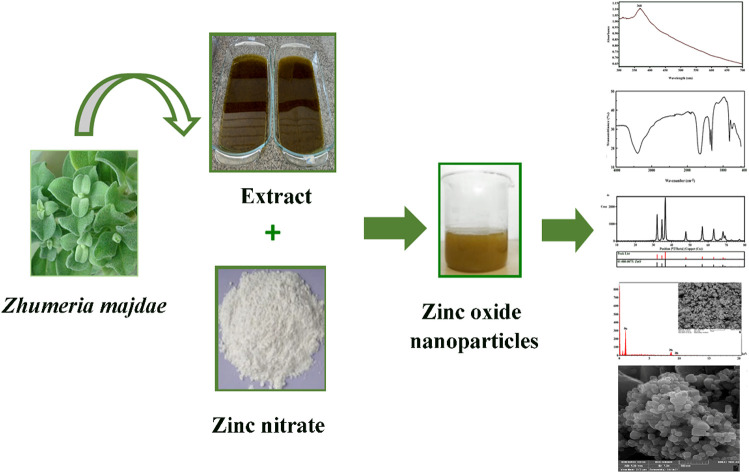

## 1. Introduction

Nanotechnology is a multidisciplinary science regarding production of materials at the nanoscale (1 to 100 nm) with promising properties such as a high surface area-to-volume, small size, charge, shape, crystal structure, thermal conductivity and surface morphology, which enables them to be used in the fields of biomedicine, and biotechnology.^1,2^ Nanoparticles (NPs) can be synthesized using chemical, physical, and biological methods. However, chemical and physical methods often produce hazardous byproducts due to the involvement of high temperatures, pressures, and toxic substances. Thus, there has been a growing interest in biological methods, also known as green nanotechnology.^3^ Green nanotechnology refers to environmentally friendly and cost-effective approaches for synthesizing NPs, which minimize or eliminate the use of hazardous materials.^4^ The green synthesis of NPs can be done using various biological sources, including bacteria, fungi, algae, and plants. Plant extracts containing compounds such as phenols, terpenoids, polypeptides, and starch serve as biological reducing agents and stabilizers in this process.^5^

Metal oxide NPs have garnered substantial attention due to their appealing characteristics. Among these NPs, zinc oxide (ZnO) NPs are particularly noteworthy because of their eco-friendly, non-toxic nature and ease of production.^6–8^ Additionally, ZnO NPs are recognized for their antibacterial and antifungal properties^9^ as well as their ability to eliminate free radicals.^10^ One of the most common and chronic diseases affecting humans and herbivorous animals is hydatidosis, also known as echinococcosis.^11^ This disease is caused by the larval stage of the *Echinococcus* parasite, which is predominantly found in regions where sheep, goats, and cattle are raised.^12^ Carnivores serve as the definitive hosts, while humans usually act as intermediate hosts in the life cycle of this parasite.^13^ The eggs of the *Echinococcus* parasite, present in the feces of infected dogs and other carnivores, are ingested by humans and herbivores. The humans are infected by vegetables, fruits or water contaminated by eggs of the cestode in the excreta of infected carnivores. The protoscolices then attach to the small intestine, where they grow and mature. The oncosphere then penetrates the intestinal wall of these intermediate hosts, is transported *via* the bloodstream, and forms hydatid cysts in the liver, lungs, spleen, kidneys, brain, muscles and, in rare cases, other organs.^14^

The substances that degrade and inactivate protoscolices include hydrogen peroxide, formalin, hypertonic saline, ethyl alcohol, and silver nitrate. These agents are associated with many side effects, such as necrosis, fibrosis, and dysfunction of infected organs including the lungs, kidneys, spleen, liver, and gallbladder.^15^ Therefore, there is an urgent need for new substances that can serve as effective protoscolicidal agents with minimal side effects. Polyphenols, phenols, and triterpenoid compounds found in plants exhibit strong antimicrobial properties against various types of bacteria, fungi, and parasites. Their diversity, abundance, and lack of side effects suggest that they may be beneficial in treating diseases caused by these organisms.^16^

The mint family, comprising 236 genera and over 7000 species, primarily thrives in temperate regions. It is widely utilized in traditional medicine due to its antimicrobial, antifungal, antioxidant, and anti-inflammatory properties. Additionally, it plays a significant role in the perfume, food, and pharmaceutical industries.^17^*Zhumeria majdae*, a species within this family, is characterized by its herbaceous, shrubby form, fragrant white or gray appearance, and blue-purple flowers, reaching a height of 50 cm. This plant is found in southern Iran and is employed in Iranian medicine to treat headaches, heart arrhythmia, and colds.^18^ The essential oil extracted from *Zhumeria majdae* is renowned for its two primary compounds: linalool (C_10_H_18_O) and camphor (C_10_H_16_O), which exhibit antimicrobial, anti-inflammatory, antioxidant, and antibacterial properties.^19^ Additionally, these compounds possess reducing and stabilizing capabilities, critical for NPs synthesis.

The green preparation of ZnO NPs using plant-based essential oils such as *Zhumeria majdae* leverages the inherent reducing and capping agents present in the oil.^20^ This approach minimizes environmental impact, offering a cost-effective and straightforward technique for producing NPs. The process typically involves mixing the essential oil with a zinc precursor, such as zinc nitrate hexahydrate. The bioactive compounds in the oil facilitate the reduction of zinc ions to form ZnO NPs. This method aligns with the growing trend of using natural sources including plant extracts, bacteria, fungi, and honey to produce NPs in an environmentally sustainable manner. The use of plant extracts introduces diverse phytochemicals including flavonoids, alkaloids, and polyphenols, which act as reducing and capping agents, influencing the size, shape, and stability of the resulting NPs. The advantages of using the *Zhumeria majdae* plant include: (1) reducing and stabilizing agent: the compounds in *Zhumeria majdae* essential oil, such as linalool and camphor, act as both reducing agents, converting zinc ions into zinc oxide, and stabilizing agents, preventing agglomeration of the NPs. (2) Eco-friendliness: green synthesis using plant extracts such as *Zhumeria majdae* essential oil reduces the need for hazardous chemicals, making the process more environmentally friendly. (3) Cost-effectiveness: plant-based synthesis is generally more cost-effective compared to traditional methods that require expensive chemical reagents and complex equipment. (4) Antimicrobial properties: *Zhumeria majdae* essential oil exhibits antimicrobial activity, which can be transferred to the synthesized ZnO NPs, enhancing their potential for biomedical applications. ZnO NPs synthesized using *Zhumeria majdae* plant have potential applications in various fields: active food packaging: the incorporation of *Zhumeria majdae* essential oil nanoemulsion into kefiran-gelatin films enhances their properties, making them suitable for active food packaging.^21^ Antimicrobial agents: these NPs can be used to control multidrug-resistant clinical pathogens, addressing the growing concern of nosocomial infections. Biomedical applications: ZnO NPs show promise in biomedicine, including wound healing, drug delivery, and antioxidant applications.^22^ Agriculture: ZnO NPs can be used to improve plant growth and development, leveraging the essential role of zinc in living organisms.^23^ Traditional chemical methods for producing ZnO NPs require high temperatures and pressures. These methods can be energy-intensive and may generate toxic byproducts. In contrast, green synthesis using the *Zhumeria majdae* plant offers a milder, more sustainable approach.

The purpose of the present study was to construct ZnO NPs from the *Zhumeria majdae* plant and to evaluate these NPs, along with the hydroalcoholic extract and essential oil, for their antibacterial, antioxidant, and scolicidal activities. *Zhumeria majdae* is utilized in the synthesis of ZnO NPs due to its unique chemical composition, eco-friendly nature, cost-effectiveness and bioactive properties, which make it a suitable candidate for green synthesis methods. Green synthesis offers an eco-friendly alternative to traditional chemical methods, which often involve hazardous reagents, complex equipment, and high energy consumption. The resulting NPs inherit beneficial properties from the essential oil, such as antimicrobial activity, making them suitable for a wide range of applications in food packaging, biomedicine, and agriculture. This green synthesis approach aligns with the growing demand for sustainable and environmentally conscious methods in nanotechnology.

## 2. Materials and methods

### 2.1. Chemicals

All chemicals, salts, and solvents required in this study were obtained from Merck with high purity. Ethanol (96%), methanol (80%), sodium acetate trihydrate (99%), acetic acid glacial (99%), 2,4,6-tri(2-pyridyl)-*s*-triazine (99%), hydrochloric acid (37%), ferric chloride (98%), ammonium iron(ii) sulfate hexahydrate (98%), 2,2-diphenyl picrylhydrazyl (95%), sodium carbonate (99.5%), folin-Ciocalteu, gallic acid (assay > 97.5%), potassium acetate (99%), aluminium chloride hexahydrate (97%), zinc nitrate hexahydrate (99%), quercetin (95%), and dimethyl sulfoxide (99.9%).

### 2.2. Collection, identification, and hydroalcoholic extraction

The aerial parts of the *Zhumeria majdae* plant (leaves, flowers, and stems) were harvested in May 2019 from an altitude of 700 meters above sea level on Mount Genu, located in Bandar Abbas city, Hormozgan Province, Iran. The plant was identified by a botanist at the Research Institute of Forests and Ranges and registered with the herbarium code TARI50023. Following collection, the aerial parts were extracted in a dry, shaded environment, using the hydroalcoholic method.^24^

### 2.3. Extraction of the essential oil analysis by GC/MS method and identification of chemical components

100 g of the aerial parts of the plant including flowers, leaves, and stems was extracted using the hydrodistillation method with a Clevenger apparatus for 3 h. The essential oil was dried with sodium sulfate (Na_2_SO_4_) and stored in a dark glass vial at 4 °C until needed.^25^ The main active compounds of the essential oil were identified using a GC-MS analysis. A Model 6890 chromatograph was coupled with an Agilent Mass Spectrometer (Model N-5973) for the qualitative analysis of the compounds. The temperature was programmed as follows: starting at 60 °C and increasing to 246 °C at a rate of 3 °C min^−1^. The injector and detector temperatures were maintained at 250 °C, with an injection volume of 1 μL and a split ratio of 1.50. Helium was used as the carrier gas, flowing at a rate of 1.5 mL min^−1^.^26^

### 2.4. Preparation and characterization of ZnO NPs

The biosynthesis of ZnO NPs was performed through the reduction of Zn(NO_3_)_2_·6H_2_O using an extract from the aerial parts of *Zhumeria majdae*. 10 mL of the filtered aqueous extract was mixed with 2 g of Zn(NO_3_)_2_·6H_2_O and heated to 60 °C while being continuously stirred at 500 rpm for 2 h. The color change from light brown to dark brown indicated the successful formation of NPs. The resulting solution was centrifuged at 12 000 rpm for 30 min and the precipitate was washed with 10 mL of double-distilled water. The obtained ZnO NPs were then placed in an oven at 100 °C for 1 h and subsequently calcined in a furnace to obtain crystalline NPs. The calcined ZnO NPs were stored in a cool, dry, and dark place.^27^ The formation of ZnO NPs was analyzed using a UV-vis spectrophotometer (UV-1900i, Shimadzu, Japan) over a wavelength range of 300 to 700 nm. The presence of phytochemical compounds and functional groups was examined through Fourier Transform Infrared (FTIR) spectroscopy (Alfa, Bruker, Germany) within the range of 4000 to 400 cm^−1^. The morphology and size of the ZnO NPs were assessed using a Field Emission Scanning Electron Microscope (FE-SEM) (Tescan Orsay Holding, Brno, Czech Republic). The presence and percentage of zinc element were determined using Energy Dispersive X-ray spectroscopy (EDS) analysis. The crystalline structure of the synthesized ZnO NPs was investigated with a Pert Power X-ray diffractometer (XRD), utilizing Cu Kα radiation over a 2*θ* range of 10 to 80°.

### 2.5. Antibacterial activity

#### 2.5.1. Determination of minimum inhibitory concentration (MIC)

The Gram-positive bacterium *Staphylococcus aureus* (ATCC 25923) and the Gram-negative bacterium *Pseudomonas aeruginosa* (ATCC 27853) were utilized, along with two antibiotics, tetracycline and imipenem as controls for the Gram-positive and Gram-negative bacteria, respectively. To determine the MIC, 100 μL of Mueller–Hinton Broth was added to each well of a 96-well plate. Subsequently, 100 μL of the hydroalcoholic extract sample (400 mg mL^−1^, DMSO), essential oil (100 μL), and ZnO NPs (0.1 g mL^−1^, DMSO) were added to the first column, with three additional replicates. Using the serial dilution method, various dilutions of the samples were prepared up to the positive control row. Finally, 100 μL of a microbial suspension equivalent to McFarland's half of 10^6^ CFU mL^−1^ was added to the wells, excluding the negative control row, and the plates were incubated for 24 h at 36 °C. The lowest clear concentration observed was defined as the MIC.^28^

#### 2.5.2. Determination of minimum bactericidal concentration (MBC) and zone of inhibition (ZOI)

To determine the MBC, 50 μL of samples from all clear wells obtained in the MIC assay was added to Mueller–Hinton agar medium and incubated at 36 °C for 24 h. The MBC is defined as the lowest concentration at which 99.9% of the bacteria do not grow. Following the MIC determination, 50 μL of sample was taken from the last clear wells and transferred to a plate containing Mueller–Hinton agar to measure the ZOI. An inoculum equivalent to 10^8^ McFarland (CFU mL^−1^) was added. It is important to note that the well method is employed for the extract, while the disc diffusion technique is utilized for the essential oil and ZnO NPs. The diameter of the inhibition zone was measured after incubation at 36 °C for 24 h.^28^

### 2.6. Antioxidant properties of the essential oil, hydroalcoholic extract and biosynthesized ZnO NPs

#### 2.6.1. Determination of phenolic and total flavonoid contents

The concentration of phenolic compounds in the hydroalcoholic extract, essential oil, and biosynthesized ZnO NPs was measured, and the results were expressed as milligrams of gallic acid equivalent per gram of dry weight of the sample. The total flavonoid content in the essential oil, hydroalcoholic extract, and biosynthesized ZnO NPs was quantified in terms of milligrams of quercetin per gram of plant material.^29^

#### 2.6.2. Determination of ferric reducing antioxidant power (FRAP assay)

The antioxidant activity of the essential oil, hydroalcoholic extract, and biosynthesized ZnO NPs was assessed, using the DPPH free radical assay method as described by Miliauskas *et al.* (2004).^30^ The capacity of the extracts to reduce Fe^3+^ ions was evaluated according to the method published by Benzie *et al.* (1996).^31^ FRAP assay is a widely used method to assess the antioxidant or reducing power of various substances, including biosynthesized ZnO NPs.^31^ The assay measures the ability of antioxidants to reduce Fe^3+^ to ferrous ions (Fe^2+^), forming a colored complex that can be quantified spectrophotometrically. The FRAP assay is based on the reduction of a ferric tripyridyltriazine (Fe^3+^-TPTZ) complex to a ferrous form (Fe^2+^-TPTZ) at low pH. This reduction is facilitated by antioxidants present in the sample. The resulting blue-colored complex is then measured by assessing the change in absorbance at a specific wavelength, typically around 593 nm. The change in absorbance is directly proportional to the reducing power of the antioxidants in the sample. FRAP values are usually obtained by comparing the absorbance change in the test reaction mixtures with those containing known concentrations of ferrous ions. The importance of FRAP assay for biosynthesized ZnO NPs includes: (1) evaluating antioxidant activity: biosynthesized ZnO NPs, particularly those synthesized using green methods, are often evaluated for their antioxidant properties. The FRAP assay provides a direct measure of the total antioxidant power of these NPs, which is crucial in determining their potential applications in biomedicine, cosmetics, and other fields. (2) Green synthesis assessment: green synthesis of ZnO NPs involves using plant extracts or other biological agents as reducing and capping agents. The FRAP assay helps to ascertain whether the resulting ZnO NPs possess enhanced antioxidant capabilities due to the bioactive compounds present in the plant extracts. (3) Comparison with other antioxidants: the FRAP assay allows for a comparison of the antioxidant power of biosynthesized ZnO NPs with that of standard antioxidants such as ascorbic acid or rutin. This comparison helps to benchmark the efficacy of the NPs as antioxidant agents. (4) Mechanism elucidation: by measuring the ferric reducing ability, the FRAP assay can provide insights into the mechanism by which ZnO NPs exert their antioxidant effects. This is particularly relevant when the NPs are used in biological systems where redox reactions play a critical role. (5) Optimization of synthesis parameters: the FRAP assay can be employed to optimize the synthesis parameters of ZnO NPs. For instance, by varying the concentration of plant extract, reaction time, or temperature, and then measuring the antioxidant activity using the FRAP assay, the optimal conditions for producing ZnO NPs with the highest antioxidant power can be determined.^32,33^

### 2.7. Scolicidal activity

#### 2.7.1. Collection of protoscolices

The liver of a sheep containing a hydatid cyst was collected from Yasouj industrial slaughterhouse in Yasouj, Kohgilouye, and Boyer Ahmad, Iran. The surface of the cysts was treated with 70% sterile alcohol, and the contents of the cysts were transferred to laboratory tubes using a sterile syringe. The samples were then centrifuged at 3000 rpm for 5 min. The resulting contents were washed three times with 0.9% sodium chloride solution and were finally stored at 4 °C until the experiment.^34^

#### 2.7.2. Scolicidal assay and viability test

To evaluate the scolicidal activity, various concentrations of the hydroalcoholic extract (25, 50, 100, and 200 mg mL^−1^), essential oil (2.5, 5, and 10 μL mL^−1^), and ZnO NPs (50, 100, 200, and 400 μg mL^−1^) were incubated for 15, 30, and 60 min. A 1 : 1 mixture of the sample and protoscolex was then prepared with 0.1% eosin and incubated for an additional 15 min. Subsequently, the mixture was placed on a glass slide, and the survival and death of the protoscolices were examined using an ordinary light microscope at 40× magnification. The live protoscolices appeared green or gray due to their selective permeability, while the dead ones were red, indicating their inability to selectively permeate and allow eosin to pass through. In this study, 9% physiological saline with Tween 80 was used as the negative control, and saturated salt served as the positive control.^35^

## 3. Results and discussion

### 3.1. Preparation of ZnO NPs

**Fig. 1:**
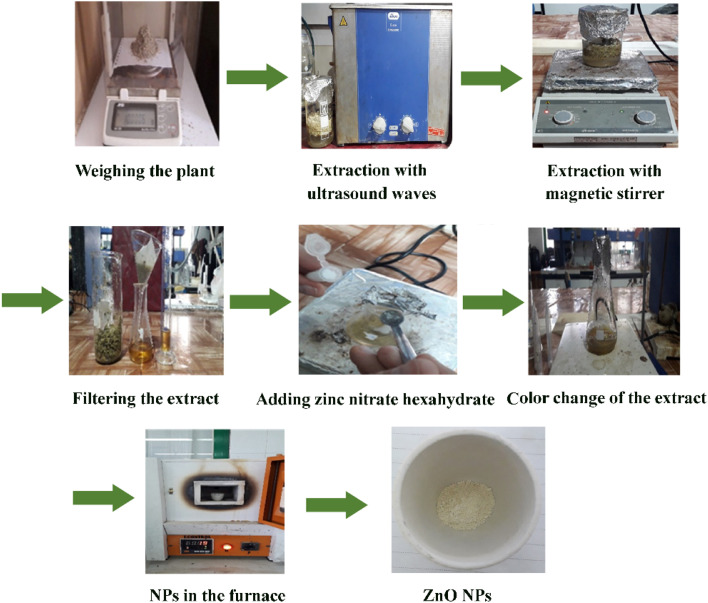
The preparation steps of ZnO NPs.

The steps for preparing ZnO NPs in the presence of the *Zhumeria majdae* plant are shown in Fig. 1.

### 3.2. Chemical composition of the *Zhumeria majdae* essential oil

**Fig. 2:**
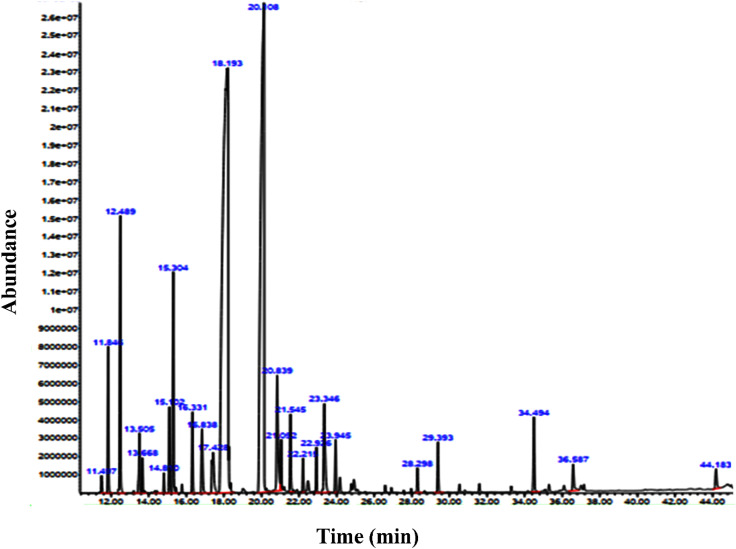
The results of spectroscopic analysis of the essential oil of the *Zhumeria majdae* plant.

The efficiency of the essential oil was recorded at 5.52% based on analytical calculations, which aligns with the findings of Saeidi *et al.* (2019), who reported an efficiency of 6.30%.^36^ GC/MS analysis was conducted to identify the chemical compounds present in the *Zhumeria majdae* essential oil, resulting in the identification of a total of 23 compounds, as listed in Table 1. Among these, three compounds exhibited the highest peak intensities (Fig. 2). The predominant components were linalool (41.50%), camphor (28.25%), and camphene (4.38%). Fallah *et al.* (2019) identified 30 compounds in the essential oil of the *Zhumeria majdae* plant, with linalool and camphor being the most concentrated, and 16 of these compounds corresponded with the current findings.^37^ Additionally, Saidi *et al.* (2019) identified 42 compounds in the essential oil of *Zhumeria majdae*, of which 18 were consistent with the present results, with linalool and camphor again showing the highest concentrations.^36^ Furthermore, according to the results of Soltani Poor *et al.*, linalool and camphor constituted the highest percentages of *Zhumeria majdae* essential oil, at 54.1% and 24.8%, respectively.^38^ Given that previous studies have already characterized the constituents of *Zhumeria majdae* using GC/MS and GC, the novelty could lie in several areas: properties of synthesized NPs, enhanced bioactivity and optimization of synthesis parameters.

The results of mass spectrometry analysis of the essential oil of the *Zhumeria majdae* plant

**Table:** 

No.	Name	RT (min)	Standard retention index	Conc. (%)	Formulation
1	α-Pinene	11.50	939	2.24	C_10_H_16_
**2**	**Camphene**	**12.48**	**953**	**4.38**	**C**_**10**_ **H**_**16**_
3	3-Octanone	13.50	993	0.97	C_8_H_16_O
4	Myrcene	13.66	994	0.47	C_10_H_16_
5	α-Terpinene	14.80	1018	0.35	C_10_H_16_
6	*p*-Cymene	15.10	1026	1.30	C_10_H_14_
7	Limonene	15.30	1031	3.70	C_10_H_16_
8	γ-terpinene	16.32	1062	1.12	C_10_H_16_
9	*cis*-Linalool oxide	16.83	1074	1.32	C_10_H_18_O
10	*trans*-Linalool oxide	17.42	1088	1.49	C_10_H_18_O
**11**	**Linalool**	**18.19**	**1098**	**41.50**	**C**_**10**_ **H**_**18**_ **O**
**12**	**Camphor**	**20.10**	**1143**	**28.25**	**C**_**10**_ **H**_**16**_ **O**
13	Borneol	20.83	1165	2.96	C_10_H_18_O
14	Terpinen-4-ol	21.05	1177	0.75	C_10_H_18_O
15	α-Terpineol	21.54	1189	1.34	C_10_H_18_O
16	Neral	22.92	1235	0.67	C_10_H_16_O
17	Geraniol	23.34	1255	2.38	C_10_H_18_O
18	Geranial	23.94	1270	0.78	C_10_H_16_O
19	*cis*-Jasmone	28.29	1394	0.42	C_11_H_16_O
20	β-Caryophyllene	29.39	1418	0.79	C_15_H_24_
21	Caryophyllene oxide	34.49	1581	1.27	C_15_H_24_O
22	β-Eudesmol	36.58	1649	0.63	C_15_H_26_O
23	Hexadecanoic acid	44.18	1984	0.47	C_16_H_32_O_2_

**Table:** 

Monoterpenes	%95
Sesqueiterpenes	%2.69
Ketone	%0.97
3-Octanone	
Palmic acid	%0.47
Hexadecanoic acid	
Alcohol *cis*-Jasmone	%0.42
Total	%99.55

### 3.3. Characterization of ZnO NPs

**Fig. 3:**
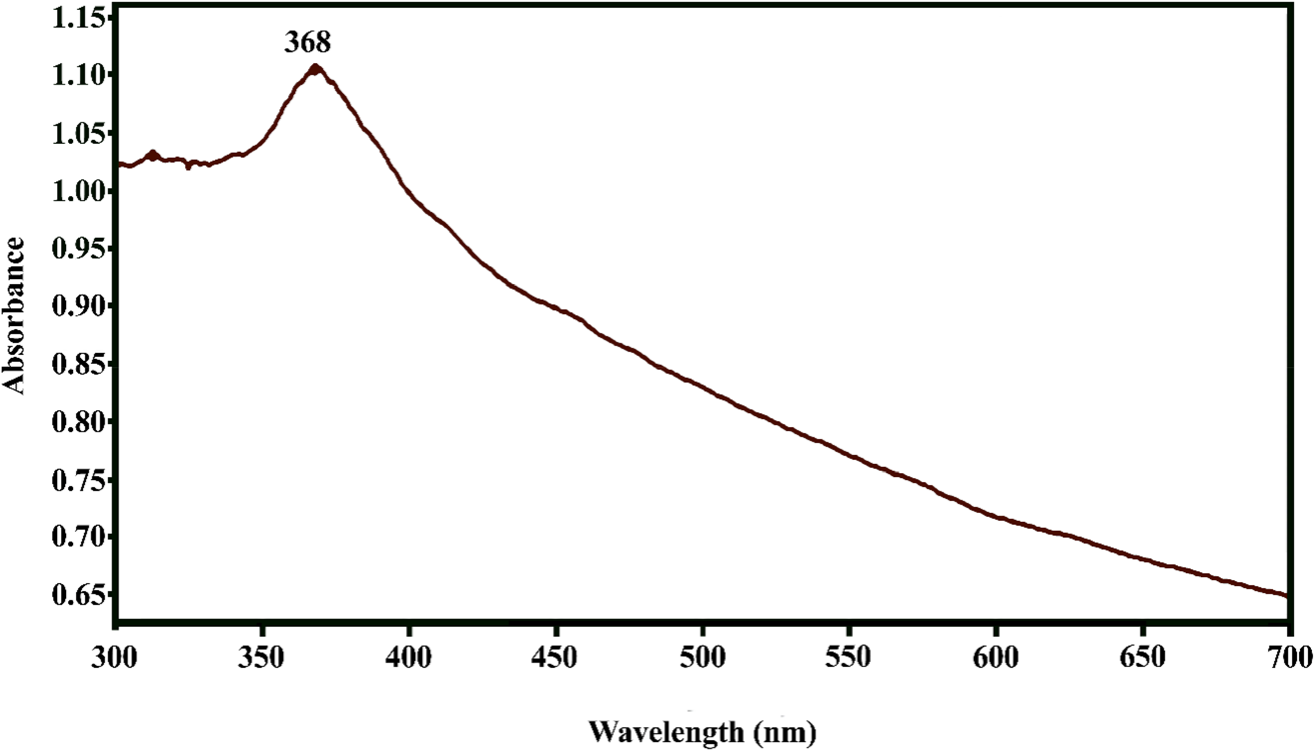
UV-vis analysis of the ZnO NPs.

Biological reduction of the metal ions in a salt solution at regular intervals leads to the formation of NPs. The color change of the solution from light brown to dark brown indicates the formation of ZnO NPs. The biological conversion of Zn^2+^ ions to Zn^0^ was mainly studied due to the presence of chemicals in the hydroalcoholic extract of the plant. The appearance of the surface plasmon resonance (SPR) band in the wavelength range of 350–400 nm confirmed the formation of ZnO NPs. The maximum absorption peak was observed at 368 nm, attributed to the SPR of ZnO NPs, indicating their formation (Fig. 3). The results of this study are consistent with those reported by Chaudhuri *et al.* (2017), Khan *et al.* (2017), and Degefa *et al.* (2021), who identified absorption peaks at 355, 347, and 375 nm for ZnO NPs, respectively.^39–41^

**Fig. 4:**
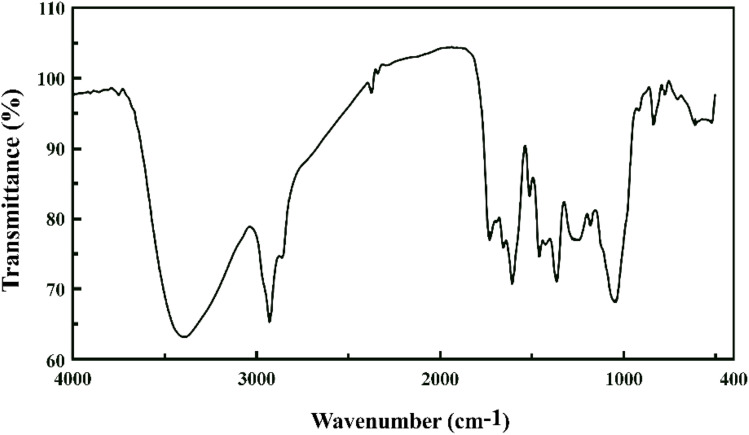
FTIR spectrum of the ZnO NPs synthesized from the *Zhumeria majdae* extract.

The determination of functional groups involved in the biosynthesis of ZnO NPs was investigated using FTIR spectroscopy (Fig. 4). The characteristic peak at 3404 cm^−1^ corresponds to the O–H groups. The peaks at 2923 and 2846 cm^−1^ represent the C–H stretching vibration mode found in alkanes. Additionally, the peak at 1730 cm^−1^ is due to the stretching vibration of C=O, and the bands at 1653 and 1602 cm^−1^ are related to the stretching vibration of C=C bonds. The peaks at 1461 and 1359 cm^−1^ contributed to bending vibrations of C–H bonds and the peak at 1051 cm^−1^ belonged to the stretching vibration of C–O. The peak at 846 cm^−1^ is associated with the stretching vibrations of ZnO synthesized by the active compounds from the plant. All these peaks indicate the presence of compounds such as flavonoids and polyphenolic phytomolecules. The results obtained from this FTIR spectrum are consistent with the findings of Basri *et al.* (2020), Ossai *et al.* (2020), and Saravanadevi *et al.* (2020).^42–44^

**Fig. 5:**
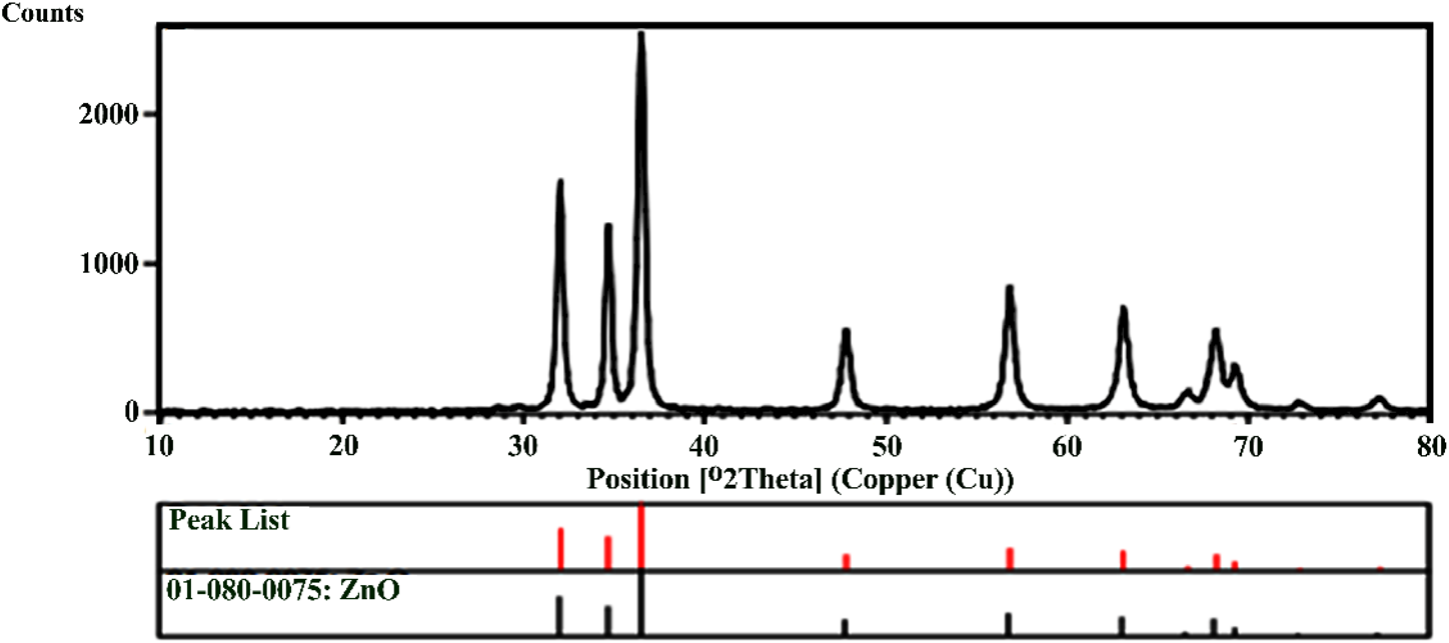
XRD pattern of the ZnO NPs.

The XRD pattern for the synthesized ZnO NPs is presented in Fig. 5. The Bragg reflection peaks observed at 2*θ* = 32.02°, 34.69°, 36.51°, 47.81°, 56.85°, 63.11°, 66.63°, 68.21°, 69.27°, 72.82°, and 77.52° are indexed as (100), (002), (101), (102), (110), (103), (200), (112), (201), (004), and (202), respectively, and correspond to the hexagonal phase of the ZnO NPs structure. The characteristic peaks show a good correlation with JCPDS standard number 01-089-1397. These results are consistent with the findings of Khaghani Boroujeni *et al.* (2018) and Abdelkhalek *et al.* (2020).^45,46^ The XRD pattern shows characteristic peaks for ZnO, indicating its crystal structure.

**Fig. 6:**
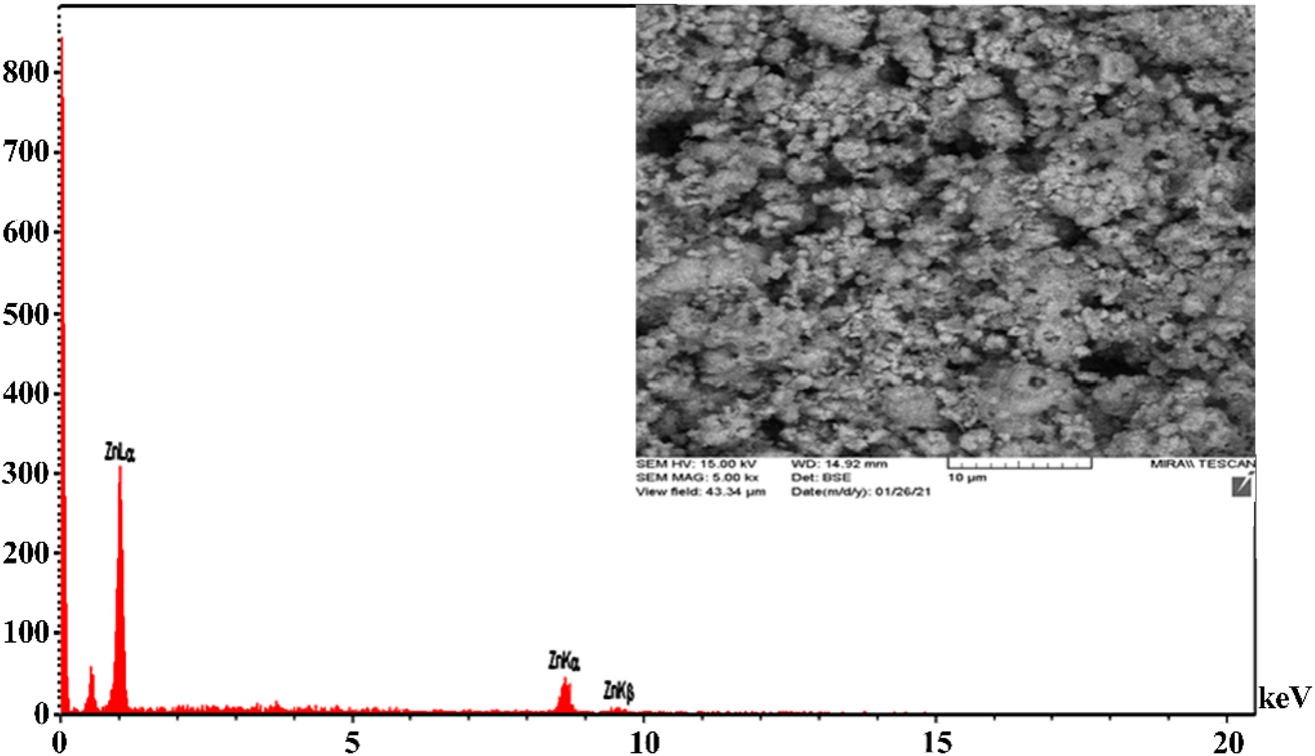
EDS of the synthesized ZnO NPs, using the *Zhumeria majdae* plant.

The chemical composition of the ZnO NPs was determined through the EDS analysis (Fig. 6). The EDS spectrum of the synthesized ZnO NPs revealed weight percentages of zinc (78.17%) and oxygen (21.8%). Additionally, the energy absorption peak for zinc was observed at 1 keV, indicating successful production of the ZnO NPs, which aligns with the findings of Rad *et al.* (2019).^47^

**Fig. 7:**
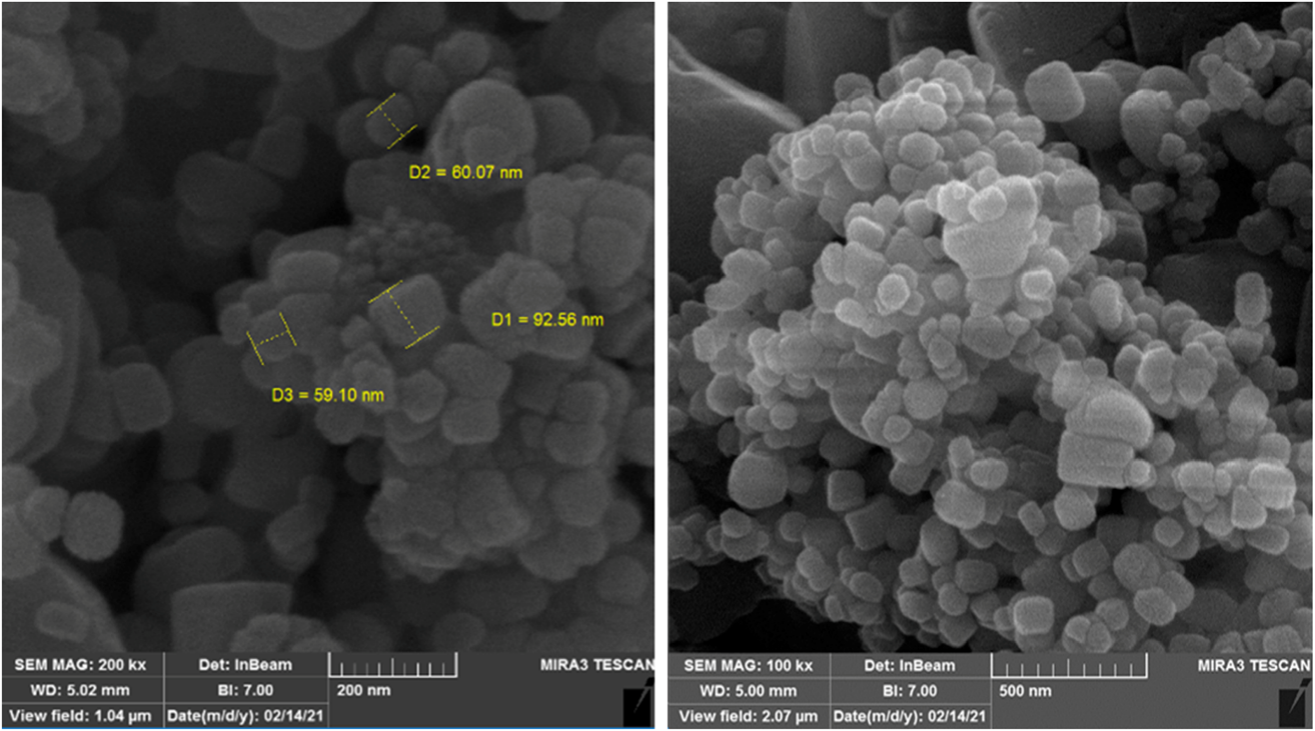
SEM ultramicrographs of the synthesized ZnO NPs from the *Zhumeria majdae* plant.

The FE-SEM ultramicrographs of the ZnO NPs are presented in Fig. 7. The images reveal cubic polyhedral NPs with sizes ranging from 69.1 to 92.6 nm. Also, some agglomeration was observed, possibly due to the presence of plant-derived organic molecules. These findings are consistent with the results reported by Suresh *et al.* (2018), Nithya *et al.* (2019), Ogunyemi *et al.* (2019) and Modwi *et al.* (2021).^48–51^ The effect of particle size on the properties of ZnO NPs is a crucial area of study due to its wide-ranging applications in various fields such as electronics, optics, catalysis, and biomedicine. The size of ZnO NPs significantly influences their physical, chemical, and biological properties, which in turn dictate their performance in specific applications. The toxicity of ZnO NPs is a significant concern, especially in biomedical applications. Smaller particles tend to exhibit higher toxicity due to their ability to penetrate cells more easily and induce oxidative stress. The release of zinc ions from the NPs can also contribute to toxicity. ZnO NPs have antibacterial properties, making them useful in antimicrobial coatings and drug delivery systems.^52^ The antibacterial activity is size-dependent, with smaller particles generally exhibiting higher activity due to their increased surface area and ability to interact with bacterial cell membranes. Smaller particles can be more easily taken up by cells, facilitating drug delivery. However, the stability and biocompatibility of these particles need to be optimized to ensure effective and safe drug delivery.

### 3.4. Antibacterial activity

**Fig. 8:**
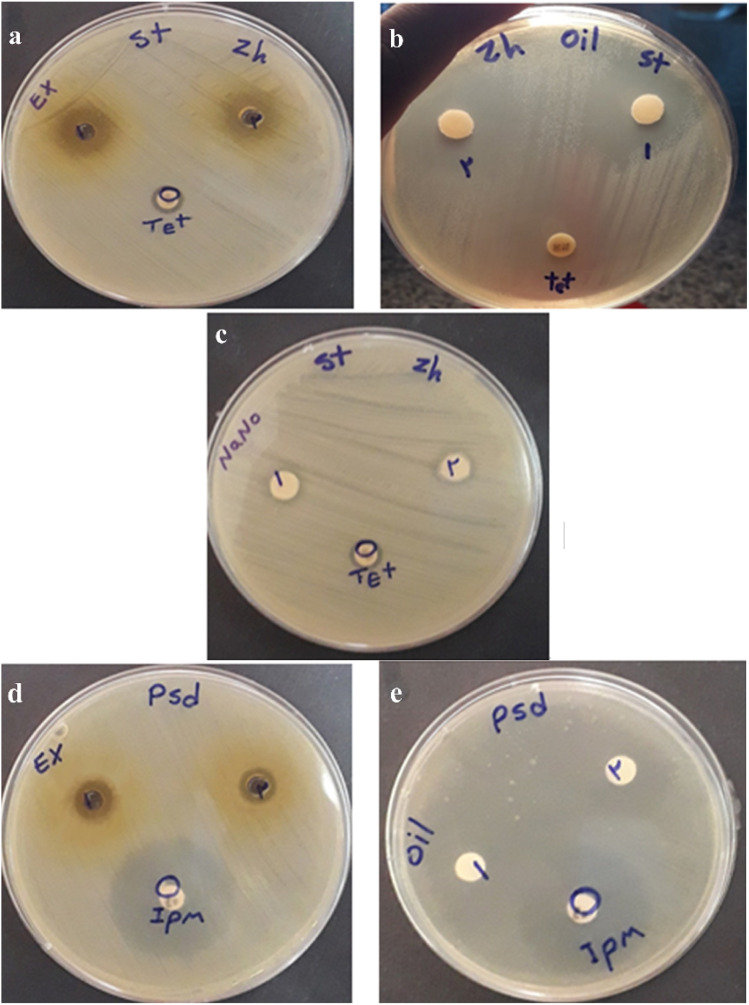
ZOI of the hydroalcoholic extract, essential oil and ZnO NPs against *Staph. aureus* (a–c), ZOI of hydroalcoholic extract and essential oil against *Pseud. aeruginosa* (d and e).

The antibacterial activity of the hydroalcoholic extract, essential oil, and ZnO NPs was investigated against the strain *Staph. aureus* and the strain *Pseud. aeruginosa* and the ZOI was measured (Fig. 8). Significant antibacterial activity was observed for *Staph. aureus*, with a MIC of 25.00 mg μL^−1^ for the hydroalcoholic extract, 3.12 μL for the essential oil, and 0.003 g μL^−1^ for the ZnO NPs. On the other hand, the minimum lethal concentration for the samples was 50.00 mg μL^−1^, 3.12 μL, and 0.003 g μL^−1^, respectively. Furthermore, the ZOI was measured 14, 31, and 7 mm, respectively, using well diffusion and disk diffusion methods. For *Pseud. aeruginosa*, the hydroalcoholic extract and essential oil exhibited MICs of 100 mg μL^−1^, and 12.5 μL, respectively. The minimum lethal concentrations were found to be 200 mg μL^−1^, and 50 μL, respectively. The ZOI for the hydroalcoholic extract and essential oil were 9 and 25 mm, respectively. This indicates that the essential oil possesses the strongest antibacterial properties against both bacterial strains, attributed to the presence of monoterpenes and terpenoids, including linalool and camphor, compared to the hydroalcoholic extract. Additionally, the ZnO NPs did not demonstrate a positive effect on *Pseud. aeruginosa* (Table 2).

**Table 2:** MIC, MBC and ZOI for different test materials against *Pseud. aeruginosa* and *Staph. aureus*

Organism	Essential oil (μL)			Hydroalcholic extract (mg mL^−1^)			ZnO NPs (mg mL^−1^)		
	MIC	MBC	ZOI (mm)	MIC	MBC	ZOI (mm)	MIC	MBC	ZOI (mm)
*Pseud. aeruginosa*	12.5	50	25	100	200	9	—	—	—
*Staph. aureus*	3.12	3.12	31	25	50	31	0.003	0.003	7

Based on the results of Table 2, the compounds present in the essential oil were correlated with the results obtained in this test. The constituents of the essential oil such as linalool, camphor, α-pinene, camphene, borneol, and α-terpineol, exhibit synergistic effects, with some demonstrating antibacterial properties. Notably, this test revealed differences in the effects on Gram-positive and Gram-negative bacteria. The cell wall of Gram-positive bacteria contains mucopeptides, while the cell wall of Gram-negative bacteria is composed of lipoproteins, lipopolysaccharides, and purines, which serve as barriers to the passage of large hydrophobic molecules, including most compounds found in the hydroalcoholic extract and the essential oil. These findings align with the results reported by Santos *et al.* (2019) regarding the sweet plant from the mint family, which showed greater efficacy against Gram-positive bacteria.^53^ Additionally, Soren *et al.* (2018) highlighted the effect of ZnO NPs on the Gram-positive bacterium *Staph. epidermidis*.^54^ Nasiri *et al.* (2014) utilized metal oxide NPs, including TiO_2_, ZnO, and CuO, to investigate their antibacterial properties. They found that *Escherichia coli* was eradicated at higher concentrations compared to *Staph. aureus*.^55^

### 3.5. Antioxidant activity

**Fig. 9:**
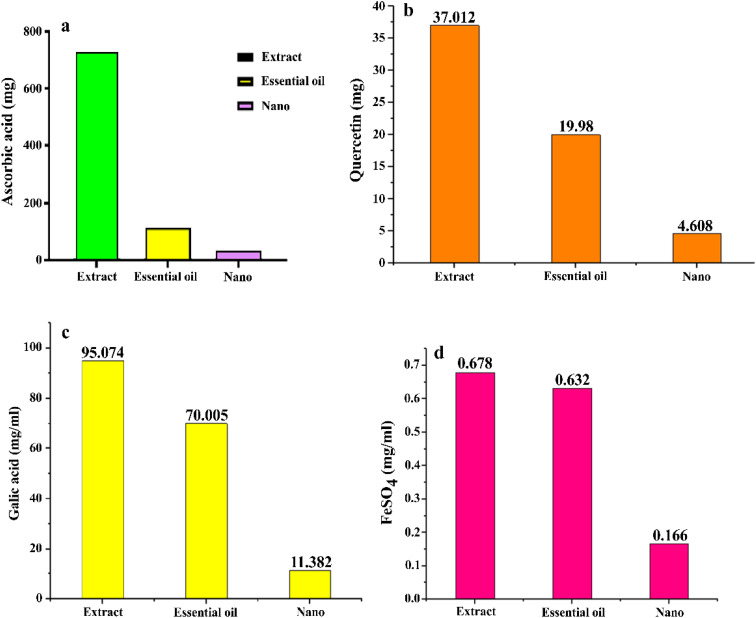
Antioxidant activity of the hydroalcoholic extract, essential oil and ZnO NPs, using DPPH (a), total flavonoid (b), total phenol (c) and FRAP (d).

Based on the DPPH test, the antioxidant activity, expressed as milligrams of ascorbic acid per gram, was highest in the hydroalcoholic extract (738.24 mg g^−1^), followed by the essential oil (118.70 mg g^−1^) and ZnO NPs (37.41 mg g^−1^). The highest concentration of total phenols, measured as milligrams of gallic acid per gram, was also found in the hydroalcoholic extract (95.07 mg g^−1^), followed by the essential oil (70.01 mg g^−1^) and ZnO NPs (11.38 mg g^−1^). Additionally, the highest amount of flavonoids, quantified as milligrams of quercetin per gram, was present in the hydroalcoholic extract (37.01 mg g^−1^), followed by the essential oil (19.98 mg g^−1^) and ZnO NPs (4.61 mg g^−1^). The highest antioxidant activity, tested by the FRAP test, was recorded for the hydroalcoholic extract (0.68 mg FeSO_4_ per g), followed by the essential oil (0.63 mg FeSO_4_ per g) and ZnO NPs (0.17 mg FeSO_4_ per g). The results of the antioxidant activity are illustrated in Fig. 9. These diagrams indicate that the antioxidant effect of the hydroalcoholic extract was superior to that of the essential oil in all the tests. The lowest level of antioxidant activity was associated with the ZnO NPs, which may be attributed to the high distribution coefficient of ethanol used as the extraction solvent. The choice of organic reagent for extracting phenolic compounds, as well as the quantity and type of the extracted compounds in terms of polarity and solubility, is directly influenced by the extraction solvent. In the study by Oalde *et al.* (2021), ethanol and water were identified as effective solvents for mint species.^56^ These findings align with those of El Aanachi *et al.* (2020), which reported a total phenol content of 106.55 micrograms per milligram of gallic acid and a flavonoid content of 49 micrograms per milligram of quercetin in the hydroalcoholic extract. This indicates a higher concentration of total phenols compared to flavonoids.^57^ Furthermore, based on the findings of Mehdizadeh *et al.* (2019) regarding the essential oil and ethanolic extract of the *Zhumeria majdae* plant, the antioxidant activity of the hydroalcoholic extract was found to be greater than that of the essential oil in all the antioxidant activity tests. This result is consistent with the findings of the present study.^58^

### 3.6. Scolicidal activity of the ZnO NPs

**Fig. 10:**
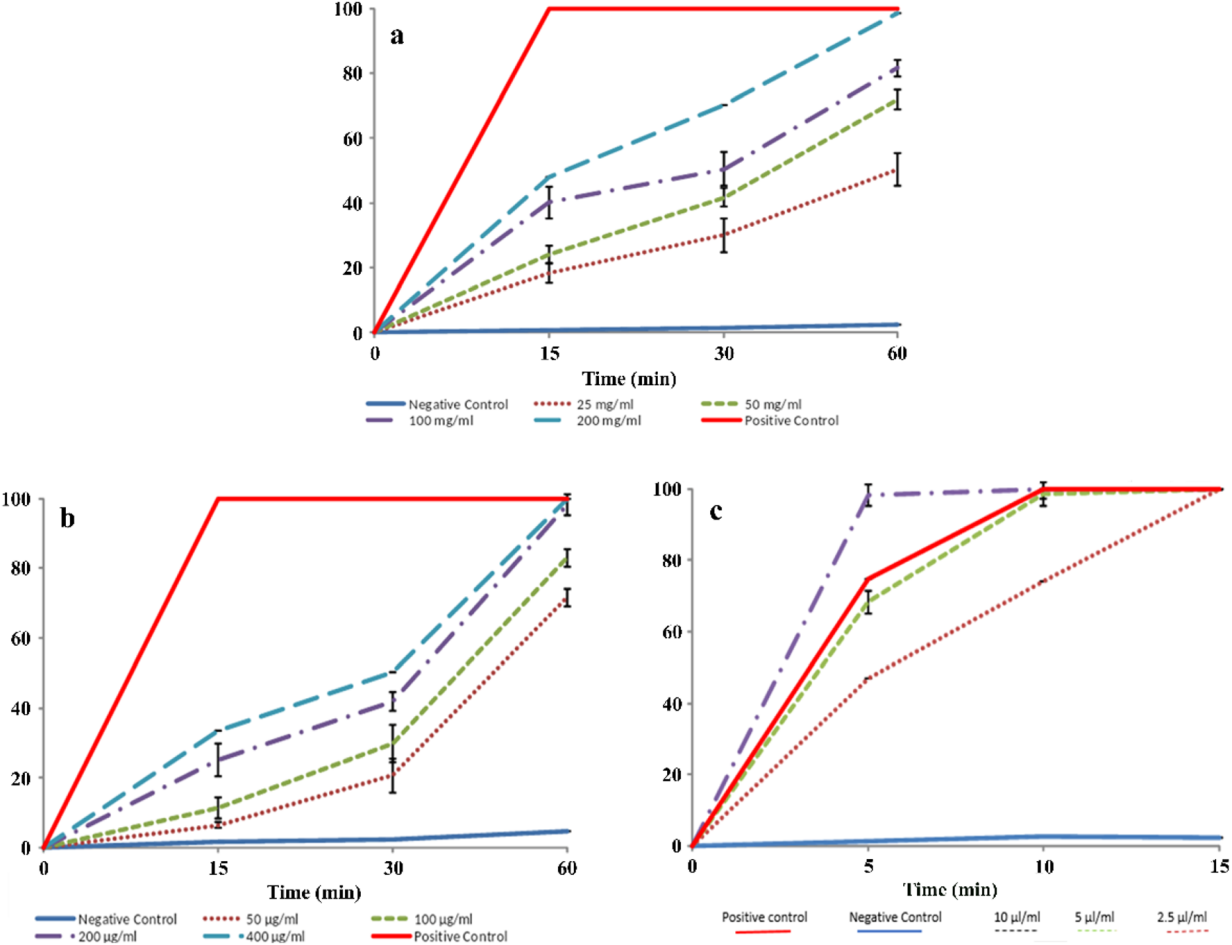
Mortality rate of protoscoleces by different concentrations and duration of exposure of hydroalcoholic extract (a), ZnO NPs (b) and essential oil (c).

**Fig. 11:**
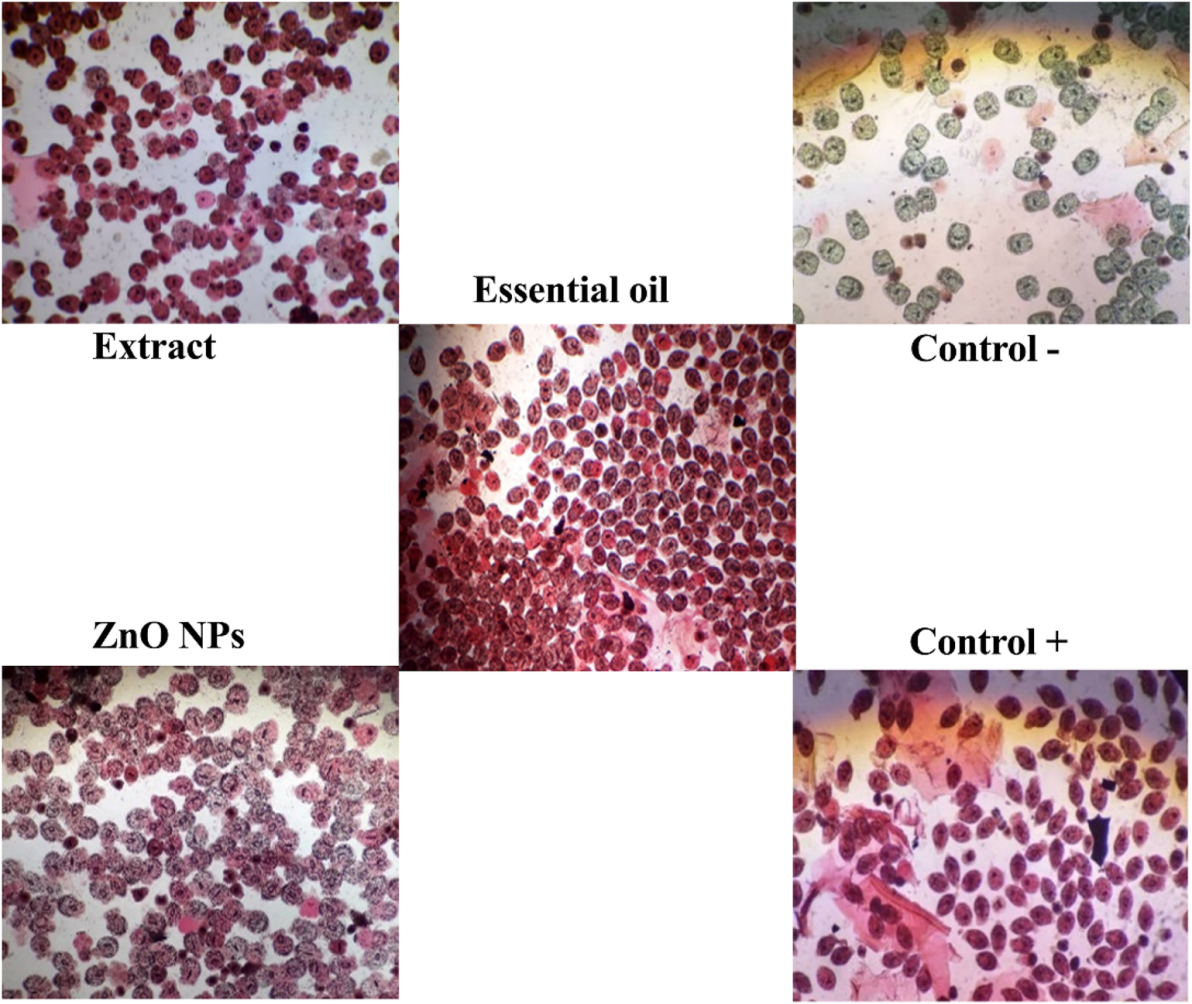
Microscopic profile of the condition of the protoscolices by hydroalcoholic extract of the *Zhumeria majdae* plant at a concentration of 200 mg mL^−1^ and exposure time of 60 min, essential oil of *Zhumeria majdae* plant at a concentration of 10 μL mL^−1^ and exposure time of 10 min, the biosynthesized ZnO NPs, using the *Zhumeria majdae* plant at a concentration of 400 μL mL^−1^ and an exposure time of 60 min and the protoscolices in the presence of positive control (saturated salt) and negative control (physiological serum + Tween 80).

As shown in Fig. 10, the highest percentage of protoscolicidal activity was observed by the hydroalcoholic extract of the *Zhumeria majdae* plant at 200 mg mL^−1^ concentration, after 60 min. Additionally, exposure to 10 μL mL^−1^ concentration of the essential oil, for 10 min, resulted in a 100% mortality rate of the protoscolices. Furthermore, the highest percentage of protoscolicidal activity from ZnO NPs was attained with a concentration of 400 μg mL^−1^ after 60 min. The survival percentage of the protoscolices, examined using a light microscope, is illustrated in Fig. 11. The average lethality percentage and standard deviation are presented in Tables 3–5.

**Table 3:** Scolicidal effect of different concentrations of hydroalcoholic extract of the *Zhumeria majdae* plant at various time intervals on protoscolices of hydatid cyst (mean ± SD)

Exposure time (min)	Concentration (mg mL^−1^)	Number of protoscolices counted	Number of dead protoscolices	Rate of dead protoscolices
15	200	503.33 ± 4.16	241.00 ± 16.37	47.87 ± 2.91
	100	508.67 ± 3.06	204.00 ± 26.32	40.09 ± 4.96
	50	503.33 ± 3.51	120.67 ± 13.65	23.97 ± 2.69
	25	501.33 ± 1.53	92.00 ± 14.73	18.34 ± 2.88
	Negative control	512	4	0.78
	Positive control	506	506	100
30	200	504.67 ± 4.16	354.33 ± 22.05	70.23 ± 4.72
	100	505.33 ± 2.52	255.00 ± 25.00	50.48 ± 5.20
	50	503.00 ± 3.00	210.00 ± 15.62	41.74 ± 2.89
	25	507.67 ± 4.04	152.33 ± 27.57	29.99 ± 5.25
	Negative control	509	7	1.38
	Positive control	508	508	100
60	200	506.67 ± 4.62	498.67 ± 16.65	98.41 ± 2.75
	100	502.00 ± 1.73	409.33 ± 13.65	81.54 ± 2.61
	50	505.67 ± 5.13	363.67 ± 10.97	71.94 ± 2.91
	25	503.00 ± 3.00	252.67 ± 23.59	50.25 ± 4.99
	Negative control	506	12	2.37
	Positive control	504	504	100

**Table 4:** Effects of different concentrations and time intervals of the essential oil of the *Zhumeria majdae* plant on the proscolices of hydatid cysts

Exposure time (min)	Concentration (mg mL^−1^)	Number of protoscolices counted	Number of dead protoscolices	Rate of dead protoscolices
5	10	502.00 ± 2.00	493.00 ± 17.35	98.2 ± 3.12
	5	504.67 ± 5.69	344.33 ± 18.48	68.22 ± 3.18
	2.5	502.33 ± 2.08	235.00 ± 14.73	46.79 ± 13.3
	Negative control	523	7	1.34
	Positive control	514	385	74.90
10	10	506.33 ± 5.69	506.33 ± 5.69	100.00 ± 0
	5	504.33 ± 3.22	496.67 ± 14.74	98.48 ± 2.64
	2.5	504.67 ± 6.43	347.67 ± 18.77	74.23 ± 3.26
	Negative control	512	13	2.54
	Positive control	507	507	100
15	10	505.67 ± 5.51	505.67 ± 5.51	100.00 ± 0
	5	506.33 ± 5.69	506.33 ± 5.69	100.00 ± 0
	2.5	503.00 ± 3.00	503.00 ± 3.00	100.00 ± 0
	Negative control	12	12	2.37
	Positive control	504	504	100

**Table 5:** Effects of different concentrations and time intervals of the biosynthesized ZnO NPs from the *Zhumeria majdae* plant on the proscolices of hydatid cysts

Exposure time (min)	Concentration (mg mL^−1^)	Number of protoscolices counted	Number of dead protoscolices	Rate of dead protoscolices
15	400	506.00 ± 6/08	169.00 ± 15/72	33/38 ± 2/79
	200	507.67 ± 4/04	127.67 ± 22.50	25.17 ± 4.63
	100	504.67 ± 2.89	57.67 ± 15.14	11.41 ± 2.93
	50	508.33 ± 7.37	32.33 ± 4.51	6.36 ± 0.83
	Negative control	507	8	1.58
	Positive control	502	502	100
30	400	507.00 ± 1.00	254.33 ± 26.00	50.16 ± 5.03
	200	506.00 ± 5.29	212.00 ± 15.62	41.89 ± 2.79
	100	510.00 ± 5.29	152.00 ± 28.05	29.81 ± 5.52
	50	510.00 ± 11.79	105.33 ± 26.10	20.62 ± 4.93
	Negative control	502	12	2.39
	Positive control	523	523	100
60	400	506.33 ± 9.29	506.33 ± 9.29	100.00 ± 0
	200	502.67 ± 3.06	494.00 ± 12.17	98.29 ± 2.97
	100	510.33 ± 5.77	423.67 ± 17.04	83.01 ± 2.62
	50	502.67 ± 4.62	360.33 ± 11.02	71.69 ± 2.54
	Negative control	502	24	4.78
	Positive control	507	507	100

Based on these results, it appears that the majority of the antimicrobial and antiparasitic activity in medicinal plants is attributed to phenolic compounds. These compounds induce degeneration and necrosis of the metacestodes by inhibiting protein or DNA synthesis and disrupting the metabolism of protoscolices. Among the factors contributing to the high antiparasitic activity of essential oils, the presence of compounds such as linalool and camphor is noteworthy. Moazeni *et al.* (2012) investigated the scolicidal effect of Khuzestani Marzeh essential oil at a concentration of 5 mg mL^−1^ over durations of 10, 20, 30, and 60 min, yielding results of 66.51%, 68.33%, 81.12%, and 100.00%, respectively. These findings indicate that the plant under study exhibits a significantly greater effect.^59^ Furthermore, Ranjber *et al.* (2020) demonstrated that the methanolic extract of *Mentha* species at a concentration of 200 mg mL^−1^ for 30 min. produced the most pronounced scolicidal effect.^60^ Additionally, Shnawa (2021) reported the scolicidal effect of ZnO NPs in the *Mentha longifolia* plant at a concentration of 400 ppm.^61^

## 4. Conclusion

The present study concluded that the phytochemical compounds found in the *Zhumeria majdae* plant serve as stabilizing and capping agents, playing a crucial role in the biological formation of ZnO NPs through an environmentally friendly, cost-effective, and straightforward process. The biosynthesized ZnO NPs were crystalline in nature and spherical, with an average size of 75 nm. The hydroalcoholic extract and essential oil derived from the *Zhumeria majdae* plant exhibited antibacterial, antioxidant, and scolicidal activities. The findings of this study showed that the essential oil of the *Zhumeria majdae* plant is more effective on some species of bacteria and also has significant scolicidal properties. These findings may have significant implications for the development of antiparasitic, antioxidant, and antibacterial drugs from the mint family. However, to achieve these objectives, further studies are necessary under *in vivo* conditions and through clinical trials.

## Author contributions

Rana Kiani: carried out the experiment, methodology, validation, investigation; Zahra Rafiee: conceptualization, data curation, visualization, supervision, writing – review & editing, project administration, funding acquisition; Damoun Razmjoue: conceptualization, data curation, writing – original draft, supervision, project administration, funding acquisition; Ahmad Oryan: data curation, writing – review & editing, visualization, Mehrorang Ghaedi: conceptualization, data curation, and Hassan Abidi: visualization, project administration.

## Conflicts of interest

On behalf of all authors, the corresponding author states that there is no conflict of interest.

## Data Availability

All data generated or analyzed during this study are included in this published article.
